# The Clinical Profile and Prognostic Factors Influencing Mortality in Patients With Acute Encephalitis Syndrome

**DOI:** 10.7759/cureus.45771

**Published:** 2023-09-22

**Authors:** Smrati Tiwari, Nikhil Ingle, Aman Goyal

**Affiliations:** 1 Internal Medicine, Seth Gordhandas Sunderdas Medical College and King Edward Memorial Hospital, Mumbai, IND

**Keywords:** prognosis, india, clinical profile, medicine, acute encephalitis syndrome

## Abstract

Introduction

Acute encephalitis syndrome (AES) is a significant global public health concern. AES is a disorder characterized by fever and altered mental status, and it is associated with considerable morbidity and mortality. There is a limited amount of existing literature on the clinical profile and prognostic markers that influence mortality in these patients. Our study seeks to comprehend the etiology, clinical characteristics, complications, and prognostic markers that impact mortality among patients with AES.

Methods

The study was a prospective observational study conducted over 18 months, involving a sample size of 105 patients. Patients aged 12 years and older, who met the WHO case definition of Acute Encephalitis Syndrome (AES), were consecutively recruited for this study. The patients' details were recorded, including their medical history and physical and clinical examination findings upon admission. The extent of cognitive impairment was evaluated using the Glasgow Coma Scale (GCS). Additionally, the patient's presenting symptoms, any complications experienced during their hospital stay, and the mortality rate were documented. The etiology, MRI results of the brain, laboratory parameters, and the need for assisted ventilation were also recorded. In-hospital characteristics were analyzed using the t-test for continuous variables and the chi-square test for binary variables. The log-rank test was employed to identify the predictors with the most significant independent influence on prognosis. All participants were selected only after obtaining their written informed consent.

Results

Most of the patients were in the age group of 21-30. 60% of the patients were male. Advanced age at presentation was associated with an increased risk of mortality (p-value=0.018). All patients presented to the hospital with symptoms of fever and altered sensorium. The most common agent isolated as the etiologic cause was *HSV-1*, found in 31.4% of the patients. 28.6% of the patients succumbed to death. The leading cause of death was raised intracranial pressure leading to hemorrhage in the brain. There was no significant correlation between the duration of symptoms and the primary outcome of death (p-value=0.498). The requirement for assisted ventilation was shown to increase the risk of death (p-value=0.001). A low GCS score at presentation was associated with a higher mortality rate (p-value=0.048).

Conclusions

The factors that predict mortality in AES involve a complex interplay of patient demographics, viral etiology, clinical severity, neuroimaging findings, and the need for assisted ventilation. Integrating these factors into clinical practice would enable healthcare providers to make informed decisions regarding patient management and interventions. As our comprehension of AES continues to develop, forthcoming advancements in diagnostics and therapeutics could refine prognostic assessments further. These developments could open new avenues for enhancing outcomes and diminishing mortality rates in this complex neurological disorder.

## Introduction

Acute encephalitis syndrome (AES) is a significant public health concern both in India and worldwide. Population-focused investigations have calculated a worldwide occurrence rate of 3.5-7.4 cases of AES cases per 100,000 individuals annually. AES is characterized by symptoms such as fever, confusion, disorientation, coma, inability to speak, and sudden onset of seizures, excluding simple febrile seizures [1]. The primary causative agent of AES in Asian nations is the Japanese encephalitis virus, which affects approximately 50,000 patients every year. Other causative viruses include Dengue, Herpes simplex virus (HSV), Varicella-Zoster, Measles, and Mumps, as well as some novel agents like Enteroviruses, Chandipura, and the Nipah virus [2].

AES is associated with substantial morbidity and mortality. However, due to the lack of a robust etiology database and an appropriate surveillance system due to poor healthcare infrastructure in third-world countries where AES is common and under-reported, standardized guidelines for prevention and management are scarce. It is essential to address the shortage of available literature on this topic. Adequate assessment of high-risk patients is crucial for early detection and subsequent management, as the case fatality rate can be as high as 30%. Additionally, permanent neurological or psychiatric sequelae can affect up to 50% of patients [2]. The effective treatment of pathogen-associated AES relies on accurate diagnosis, which can be challenging because of the wide range of etiological agents responsible for causing similar clinical manifestations [3]. Currently, treatment primarily involves supportive management, ensuring airway maintenance, adequate breathing, and sufficient circulation. Definitive treatment with antiviral agents may be necessary for specific patients based on their etiology [3].

Our study aims to comprehend the etiology, clinical features, complications, and prognostic markers that influence mortality in patients with AES.

## Materials and methods

Study design  

This prospective observational study was conducted over a period of 18 months, from June 2019 to December 2020, in a sample size of 105 patients. The study was conducted in the Department of Internal Medicine at a tertiary care hospital in Mumbai, India. 

Inclusion criteria 

Individuals aged ≥ 12 years who met the WHO Health Organization criteria for AES were enrolled in this study. According to the WHO Organization’s case definition, AES is characterized by the presence of fever or a recent history of fever coupled with alterations in mental status (such as confusion, disorientation, coma, or inability to communicate) and/or the onset of new seizures (excluding simple febrile seizures) [4]. 

Exclusion criteria 

Patients who exhibited altered mental status due to metabolic abnormalities, including hepatic encephalopathy, uremic encephalopathy, and hypoglycemia, were excluded from the study. Similarly, individuals diagnosed with tuberculous meningitis or cryptococcal meningitis were also excluded. Patients whose acute confusion state had a proven psychiatric etiology, as well as those whose condition was attributed to drug-induced factors, were not included in the study cohort. Additionally, individuals with autoimmune causes and those diagnosed with cerebrovascular accidents were excluded from the research analysis.

Study procedure 

The patients' details were recorded, including their medical history as well as the findings from their physical and clinical examinations upon admission. The Glasgow Coma Scale (GCS) was used to assess the extent of cognitive impairment in the patients during their initial presentation. Its role in independently determining patient prognosis has been investigated. Additionally, the patient's presenting symptoms, presence of seizures at the time of presentation, any complications experienced during their hospital stay, neuroschiatric sequelae at the time of discharge, and mortality rate were documented. The primary outcome was patient death. Other variables studied included the etiology of the condition, MRI results of the brain, laboratory parameters, and the need for assisted ventilation.

Statistical analysis 

In-hospital characteristics were analyzed using the t-test for continuous variables and the chi-square test for binary variables. Age was adjusted for mortality to determine the existence of any correlations. The log-rank test was used to identify those predictors with the most significant independent influence on prognosis. All p-values less than 0.05 were considered statistically significant with a confidence interval of 95%. All statistical analyses were performed with the use of the IBM SPSS version 28 (IBM Corp., Armonk, USA). 

Ethical considerations

Ethical approval was granted by the Institutional Ethics Committee (IEC-II) of Seth Gordhandas Sunderdas Medical College and King Edward Memorial Hospital. The approval number was EC/10/2018. Furthermore, this study followed the principles of the Declaration of Helsinki. All participants were only selected after taking their written informed consent. 

## Results

Demographic information 

Most of the patients (48%) were in the age group of 21-30 years. 60% of the patients were male (Table 1). Advanced age at presentation was correlated with an increased risk for mortality (p-value=0.018). 

**Table 1:** The distribution of demographic variables

Variable	Frequency (n=105)
1-Age distribution (in years)	
<20	18 (17.1%)
21-30	48 (45.7%)
31-40	15 (14.3%)
41-50	12 (11.4%)
51-60	9 (8.6%)
>60	3 (2.9%)
2- Gender	
Males	63 (60%)
Females	42 (40%)

Etiology 

The most frequently isolated agent identified as the etiological cause was HSV-1, which was found in 31.4% of patients. This was followed by dengue encephalitis, which was observed in 20% of the patients. The etiology remained undetermined in 37.1% of the patients (Table 2). The primary causes of undetermined etiology were inconclusive test results and unavailability of testing. 

**Table 2:** The etiology of acute encephalitis syndrome HSV: Herpes simplex virus; EBV:

Etiology	Frequency (n=105)
Undetermined causes	39 (37.1%)
HSV 1-Encephalitis	33 (31.4%)
Dengue Encephalitis	21 (20.0%)
HIV encephalitis	9 (8.6%)
EBV encephalitis	3 (2.9%)

Clinical profile and course in the hospital 

All patients (n=105) presented to the hospital with symptoms of fever and altered sensorium. Headache, seizures, and vomiting were other frequently encountered symptoms in the patients. Seizures were seen in 91.4% of the patients and were both generalized and focal in origin. 66.6% of the patients had no complications during their stay in the hospital. The most frequently encountered complication was multi-organ dysfunction seen in 17.1% of the patients (Table 3). 71.4% of the patients were successfully discharged from the hospital, while 28.6% of the patients, unfortunately, succumbed to death. At the time of discharge, 20% of the patients had neuropsychiatric sequelae present. The most common cause of death was raised intracranial pressure causing hemorrhage into the brain seen in 60% of the patients (Table 3). Brain MRI was performed at the time of admission, and 74.3% of the patients had abnormalities detected, consistent with the underlying pathological process (Table 4). Among the laboratory parameters, the mean hemoglobin levels were 11.5 g/dL with an elevated erythrocyte sedimentation rate (ESR) of 37.7 mm/hour. The mean magnesium levels were low at 1.2 mg/dL. The mean cerebrospinal fluid (CSF) protein levels were elevated with a value of 99.0 mg/dL and the mean cerebrospinal fluid glucose levelswere slightly low. The lymphocyte levels in the CSF were also elevated. The liver and renal function tests were within normal limits (Table 5). 

**Table 3:** Clinical profile and course in the hospital

Variable		Frequency (n=105)
1- Presenting signs and symptoms		
Altered sensorium		105 (100.0%)
Seizures		96 (91.4%)
Fever		105 (100.0%)
Headache		84 (80.0%)
Vomiting		57 (54.2%)
2-Type of seizure at presentation		
Generalized tonic-clonic		81 (77.1%)
Focal with secondary generalization		9 (8.6%)
Focal		6 (5.7%)
3-Complications seen		
No complication		66 (62.9%)
Acute kidney injury		9 (8.6%)
Hepatitis		9 (8.6%)
Multi-organ dysfunction		24 (17.1%)
Aspiration Pneumonitis		3 (2.8%)
4- Primary outcome		
Discharge	Without Neuropsychiatric sequelae	54 (51.4%)
	With neuropsychiatric sequelae	21 (20.0%)
Death		30 (28.6%)
5-Neuropsychiatric sequelae at the time of discharge		
No neuropsychiatric sequelae		84 (80.0%)
Global cognitive decline		6 (5.7%)
Anterograde amnesia episodic memory loss		12 (11.4%)
Psychosis		3 (2.9%)
6-Cause of Death		(n=30%)
Raised intracranial pressure causing brain hemorrhage		18 (60.0%)
AKI with metabolic acidosis		6 (20.0%)
Aspiration Pneumonitis		3 (10.0%)
Hepatitis with acute liver failure		3 (10.0%)

**Table 4:** Brain MRI findings observed in the patients

1-Brain MRI findings	Frequency (n=105)
Normal MRI with no abnormalities	27 (25.7%)
T2 hyper-intense lesion with restricted diffusion in the frontoparietal lobe	24 (22.8%)
T2 hyper-intense lesions with restricted diffusion in the temporal lobe	27 (25.7%)
T2 hyper-intense lesions in bilateral hippocampus	6 (5.7%)
T2 hyper-intense lesions with restricted diffusion in the frontal lobe	3 (2.9%)
T2 hyper-intense lesions with restricted diffusion in the bilateral thalamus	24 (17.1%)

**Table 5:** Mean laboratory parameters observed in the patients The number following ± indicates the standard deviation value ESR: Erythrocyte sedimentation rate; BUN: Blood urea nitrogen; SGPT: Serum glutamic pyruvic transaminase; SGOT: Serum glutamic oxaloacetic transaminase

Variable	Mean Value
1-Hematological indices	
Hemoglobin (g/dL)	11.5 ± 2.37
WBC (per mm3)	8890.0 ± 3033.50
Platelet count (/mm3)	229000 ± 0.92
ESR (mm/hour)	37.7 ± 14.23
2-Renal function tests	
BUN (mg %)	12.5 ± 5.79
Serum creatinine (mg %)	1.1 ± 0.31
3-Liver function tests	
SGOT (U/L)	35.5 ± 18.0
SGPT (U/L)	22.9 ± 13.7
Total bilirubin (mg %)	0.7 ± 0.2
Direct bilirubin (mg %)	0.14 ± 0.1
4-Serum electrolyte concentrations	
Sodium (mEq/L)	136.1 ± 3.7
Potassium (mEq/L)	3.8 ± 0.42
Chloride (mEq/L)	100.8 ± 5.3
Calcium (mg/dL)	8.8 ± 0.31
Magnesium (mg/dL)	1.2 ± 0.1
5- Cerebrospinal fluid parameters	
Proteins (mg/dL)	99.0 ± 3.2
Glucose (mg/dL)	46.9 ± 4.3
RBC count (per mm3)	36.6 ± 2.1
Polymorphs (per mm3)	1.3 ± 0.3
Lymphocytes (per mm3)	28.6 ± 1.3

Prognostic markers for morbidity and mortality 

The mean duration for which symptoms lasted in the patients was 5.69 days with the minimum duration being three days and the maximum being 10 days. There was no significant correlation between the duration of symptoms and the primary outcome (p-value=0.498). Of the 105 patients studied, only 39 required assisted ventilation. Out of those 39 patients, 30 patients succumbed to death. 66 patients did not require assisted ventilation and all those patients survived (Table 6). The requirement for assisted ventilation correlated with a higher mortality rate (p-value=0.001). This may be partly due to the need for ventilation being higher among patients who present late in the disease course and thus have a worse prognosis. In patients with a GCS score of less than or equal to 8, accounting for 18 patients, 12 patients succumbed to death, and only six survived. Out of those having a GCS score of more than 8, accounting for 87 patients, 69 patients survived and 18 patients succumbed to death (Table 6). A low GCS score at presentation was correlated with higher mortality (p-value=0.048).  

**Table 6:** Association between assisted ventilation and GCS score at presentation with the primary outcome GCS: Glasgow Coma Scale

Variable		Number of patients with discharge as the outcome	Number of patients with death as the outcome
1-Requirement for assisted ventilation	Yes (n=39)	9	30
	No (n=66)	66	0
2- GCS score at presentation	Less than or equal to 8 (n=18)	6	12
	Greater than 8 (n=87)	69	18

## Discussion

In our study, 37.1% of patients with acute encephalitis syndrome had undetermined etiology. In another similar study, despite extensive testing, the etiologies of over three-fourths of the cases remained unknown [5,6]. New, cost-effective strategies for pathogen identification could enhance our ability to diagnose encephalitis and improve outcomes.

The prognosis of AES is grim. In our study, 28.6% of patients succumbed to the illness, while 20% had neuropsychiatric sequelae upon discharge. Among patients discharged with neuropsychiatric manifestations, six developed global cognitive decline, 12 experienced anterograde amnesia with episodic memory loss, and three developed psychoses. Comparable studies by Poneprasert B et al. and Kumar et al. reported high mortality rates of 17% and 30%, respectively, along with neurological sequelae as high as 57% in Poneprasert B et al.'s study [7,8]. Our research contributes further evidence that AES is a deadly condition. Hence, it is crucial for physicians to identify factors that increase the risk of mortality and morbidity in these patients. Physicians should closely monitor factors such as the need for assisted ventilation, advanced age, and a Glasgow Coma Scale score below 8 at presentation. Similar studies by Kakoti et al. have indicated that children with GCS equal to or less than 8 and the presence of meningeal irritation had significantly higher fatality rates [9,10]].

Moreover, neuroimaging studies, particularly magnetic resonance imaging (MRI), provide valuable insights into the extent of brain involvement. They can also offer diagnostic clues regarding etiology based on the affected brain area. Imaging showing diffuse cerebral edema, multiple lesions, and brainstem involvement is associated with increased mortality [11]. Examination of cerebrospinal fluid (CSF) can aid in prognostication. Elevated white blood cell count, increased protein levels, and the presence of viral RNA in CSF are indicators of a more severe disease course [6].

Our study had certain limitations. Firstly, the definition of AES is clinical and subject to interpretation. This is likely to result in the over-inclusion of non-encephalitic cases such as AES. However, this is an operational definition, and it is difficult to have a high degree of accuracy to define true encephalitis. Secondly, the risk of disease incidence and the hazard of mortality or disability are likely to be etiology-specific. Previous studies have indicated that AES can be attributed not only to a single virologic agent, such as the Japanese encephalitis virus (JEV) but also to a myriad of viruses. The most common agent in India is JEV, while other studies have shown an increasing incidence of non-JEV etiologies in recent years [11-12]. Other viruses contributing to AES include Dengue Virus, HSV, measles virus, mumps virus, Varicella Zoster virus, Chandipura virus, and enteroviruses [12-15]. However, this limitation is not unique to our study; it is a challenge faced by many similar studies. Lastly, it's important to note that all patients included in this study were recruited from a single tertiary hospital, which may not accurately represent the entire population.

Considering the broad range of causal agents and the rapid onset of neurological impairment due to pathogenesis, clinicians are confronted with the challenge of a narrow window period between diagnosis and treatment. A robust surveillance program and improvements in the monitoring system are necessary. Timely reporting of fever cases accompanied by altered mental status to a disease surveillance system would be advantageous for determining aetiological trends. Given that many cases of AES still lack a known etiology [16], this approach could contribute to the identification of specific causes for future treatment options.

## Conclusions

In conclusion, we emphasize the importance of early identification of AES in high-risk groups, such as individuals of advanced age who present with low GCS scores and require ventilation. This emphasis encourages physicians to closely focus on this demographic to improve outcomes. Collaborative efforts among clinicians, researchers, and public health officials are imperative for enhancing prognosis accuracy and patient outcomes in AES. As our understanding of AES continues to evolve, upcoming advancements in diagnostics and therapeutics could further refine prognostic assessments. These developments might open new avenues for improving outcomes and reducing mortality rates in this intricate neurological disorder.
